# Polymorphism in the symmetries of gastric pouch arrangements in the sea anemone *D. lineata*

**DOI:** 10.1186/s40851-021-00180-0

**Published:** 2021-09-06

**Authors:** Safiye E. Sarper, Tamami Hirai, Take Matsuyama, Shigeru Kuratani, Koichi Fujimoto

**Affiliations:** 1 Department of Biological Sciences, Graduate School of Science, Osaka University, Toyonaka, Osaka, 560-0043 Japan; 2 Laboratory for Evolutionary Morphology, RIKEN Center for Biosystems Dynamics Research (BDR), Kobe, 650-0047 Japan; 3 Laboratory for Retinal Regeneration, RIKEN Center for Biosystems Dynamics Research, Kobe, 650-0047 Japan; 4 Evolutionary Morphology Laboratory, RIKEN Cluster for Pioneering Research (CPR), Kobe, 650-0047 Japan

**Keywords:** Phenotypic variation, Symmetry, Cnidaria, Anthozoa, Gastric pouch

## Abstract

Symmetry in the arrangement of body parts is a distinctive phylogenetic feature of animals. Cnidarians show both bilateral and radial symmetries in their internal organs, such as gastric pouches and muscles. However, how different symmetries appear during the developmental process remains unknown. Here, we report intraspecific variations in the symmetric arrangement of gastric pouches, muscles, and siphonoglyphs, the Anthozoan-specific organ that drives water into the organism, in *D. lineata* (Diadumenidae, Actiniaria)*.* We found that the positional arrangement of the internal organs was apparently constrained to either biradial or bilateral symmetries depending on the number of siphonoglyphs. Based on the morphological observations, a mathematical model of internal organ positioning was employed to predict the developmental backgrounds responsible for the biradial and bilateral symmetries. In the model, we assumed that the specification of gastric pouches is orchestrated by lateral inhibition and activation, which results in different symmetries depending on the number of siphonoglyphs. Thus, we propose that a common developmental program can generate either bilateral or biradial symmetries depending on the number of siphonoglyphs formed in the early developmental stages.

**Supplementary Information:**

The online version contains supplementary material available at 10.1186/s40851-021-00180-0.

## Introduction

**Fig. 1:**
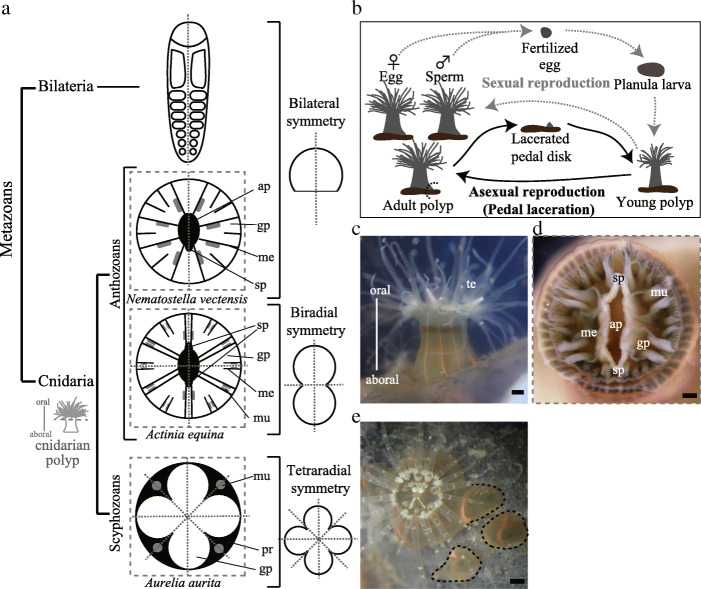
Body symmetries on metazoan phylogeny. **a** Schematic diagram of a bilaterian embryo and three representative cnidarian polyps with different symmetry types. A section of cnidarian polyps (gray dashed line) perpendicular to the oral–aboral axis representing the positional arrangement of gastric pouches. Symmetry planes of these arrangements are represented by gray dotted lines. **b** Schematic of life cycles for sexual and asexual reproduction (pedal laceration) modes. **c** External view of *D. lineata*. Orange-striped patterns orient to the oral–aboral axis in a longitudinal green body column. Scale bar, 2 mm. **d** Internal view of *D. lineata.* Horizontal cut of a polyp showing the actinopharynx, siphonoglyph, muscles, and mesenteries. Scale bar, 200 μm. **e** Asexual reproduction of a few individuals (framed with black dashed lines) by pedal laceration. Scale bar, 1 mm. ap: actinopharynx, gp: gastric pouch, sp: siphonoglyph, mu: muscle, me: mesentery, pr: partition, te: tentacles

Symmetry in the arrangement of external and internal organs is a distinctive phylogenetic feature of animals [1–5]. Bilaterians (e.g., vertebrates) are defined by bilateral symmetry, in which paired organs are arranged in a mirror image to a single symmetry plane (Fig. 1a). Cnidarians represent a phylum generally described by radial symmetry, with more than one symmetry plane. In cnidarian polyps, symmetrical arrangement is apparent in internal organs such as gastric pouches and muscles that arise perpendicular to the oral–aboral axis (Figs. 1a, c, d) [6, 7]. Tetraradial symmetry is identified by four symmetrical planes. In scyphozoans (e.g., *A. aurita*), tetraradial symmetry results in four gastric pouches roughly separated via partitions with four muscles arranged in a mirror image [8] (Fig. 1a). Interestingly, in cnidarians, there are some lineages that show bilateral symmetry [1, 4, 5, 9–11] (Fig. 1a). However, how these different symmetries appear remains unknown.

Both biradial and bilateral symmetries are found in anthozoan species [7, 12] (Fig. 1a). In these animals, gastric pouches develop as the result of the sequential development of mesenteries that separate gastric cavities [13–15] (‘me’ in Fig. 1a). The mesenteries extend from the body wall to the actinopharynx (‘ap’ in Fig. 1a), a tube-like organ at the center of the oral surface (Figs. 1a, d). Retractor muscles (‘mu’ in Fig. 1a) are attached on either side of the mesenteries. In addition to the mesenteries and muscles, there are siphonoglyph organs placed at longitudinal termini of the actinopharynx to control the water current inside the organism (‘sp’ in Fig. 1a, d). Species with bilateral symmetry in the gastric pouch and muscle arrangements mostly have a single siphonoglyph on the single symmetry plane (Fig. 1a). Some other species showing biradial symmetry possess two siphonoglyphs, with one symmetry plane that passes through the longitudinal axis of the actinopharynx and the other perpendicular to it (Fig. 1a). Although the correlation between the types of symmetries and the number and positions of siphonoglyphs is widely evident among anthozoans [12, 16], whether and how siphonoglyphs control the symmetric arrangement of gastric pouches and muscles remains unclear.

Internal organs that can be employed as symmetry indicators, such as gastric pouches, muscles, and siphonoglyphs, exhibit intraspecific variations in their arrangement and number in many anthozoan species [17, 18]. Nevertheless, whether there is intraspecific variation in symmetries remains elusive. Analyzing the developmental processes of such symmetry variation in a single species sharing the same genetic toolkit could provide a developmental background for different symmetries.

To examine the variations in symmetry, *D. lineata* (Actiniaria, Metridioidea, and Diadumenidae; Verrill 1869) is an ideal candidate for an experimental model because it exhibits high levels of variation in its gastric pouches, muscles, and siphonoglyphs [17]*. D. lineata* was originally described in Japan and is widely distributed along the shores of Japan, Europe, and North America [19–21]. Commonly among sea anemones, both sexual and asexual reproduction appear in *D. lineata* (Fig. 1b). Sexual reproduction begins when eggs and sperm are released from adult polyps. The fertilized eggs develop into free-swimming larvae known as planula [22], which later attach to the substratum and then transform into polyps (Fig. 1b). To the best of our knowledge, sexual reproduction of *D. lineata* has not been reported in nature, and previous laboratory experiments have failed to successfully induce sexual reproduction with nonattached planulae [22]. Asexual reproduction (pedal laceration) starts with the asymmetric fission of a body part at the aboral side, referred to as the pedal disk, which eventually regenerates into new polyps [17] (Fig. 1b). The polyps exhibit orange-striped pigmentation along the walls of their gastric pouches (Fig. 1c), whose numbers vary between 6 and 18 with an average of 12, whereas those of siphonoglyphs vary between one and four [17]. These variations in the number were proposed to be ascribed to the internal organ variations that appear at the detached pedal disk, which further reproduce new internal organs by asexual reproduction (Fig. 1b, e) [17].

Here, we show a polymorphism of bilateral and biradial symmetries in the gastric pouches and muscles of *D. lineata.* Bilaterally symmetric arrangements were mainly seen in individuals with a single siphonoglyph, whereas biradially symmetric arrangements were found in individuals with two siphonoglyphs, revealing a correlated variation between the symmetry type and the siphonoglyph number. Based on the observed arrangements, we built a mathematical model of the fate specification of gastric pouches during the asexual reproduction process. This model predicted that a common developmental program could potentially produce both bilateral and biradial symmetries. The symmetry difference was encoded by the siphonoglyph number at the initial stage of asexual reproduction in *D. lineata*.

## Materials & methods

### Sample collection and nursery

**Fig. 2:**
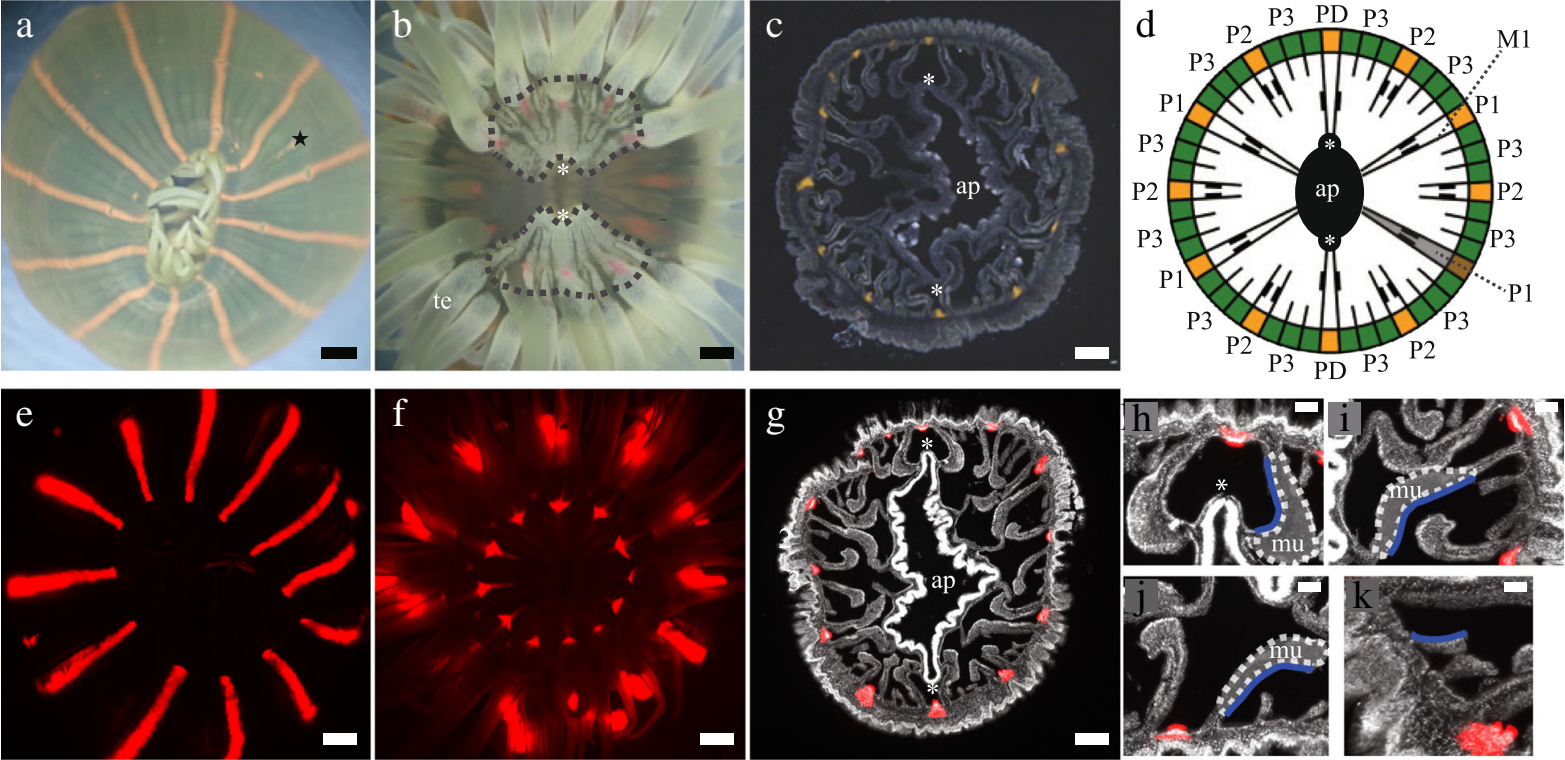
Morphology and organ arrangement of *D. lineata.* **a** External view of a *D. lineata* polyp*.* Transparency of the ectodermal cell layer allows us to see black mesenteries and orange-pigmented stripes at PDs, P1s, and P2s, while orange pigmentation sporadically appears at P3s (star). Scale bar indicates 1 mm. **b** Longitudinal actinopharynx and siphonoglyphs located on the oral disk surrounded by tentacles. Fan-shaped white pigmentation (outlined with gray dashed lines) appears at the base of the tentacles centered around the siphonoglyph. Scale bar indicates 1 mm. te: tentacles. **c** Horizontal histological section demonstrating orange pigmentation at the endodermal cell layer of the PD, P1, and P2 walls. Scale bar, 200 μm. **d** Schematic view of a horizontal section of a biradially symmetrical 12-striped (orange) individual. Green regions indicate P3s and pouches adjacent to P3s. Orange regions indicate P1s, P2s, and PDs. **e**, **f** External view of red fluorescence protein colocalized with orange stripes at (**e**) the root of tentacles on the oral disk (**f**). Scale bar indicates 1 mm. **g** Horizontal histological section demonstrating mesentery, muscle, and siphonoglyph arrangement. Nuclei are labeled with 4′,6-diamidino-2-phenylindole (white). Scale bar indicates 200 μm. **h–k** Enlarged views of histological sections of G showing a (**h**) MD, (**i**) M1, (**j**) M2, and (**k**) M3 (white dotted lines indicate the outline of muscle, blue lines indicate the mesenteries). Scale bars, 50 μm. ap: actinopharynx, mu: muscles, M1: first mesentery. PD, P1, P2, and P3 denote directive, first, second, and third gastric pouches, respectively. Asterisks indicate siphonoglyphs

All *D. lineata* samples (Fig. 2) were collected at Jougasaki and Isonoura, Wakayama prefecture, Japan, during the low tide cycle between March and December 2019 using forceps to gently detach the pedal disk from the substrate. In the laboratory, anemone samples were kept in artificial seawater at room temperature (23–27 °C) with a 12-h day/night cycle and fed *A. salina* (brine shrimp) once a week throughout the experimental process.

### Organ arrangement and anatomical definitions

In previous studies, symmetry types of anthozoan species were mostly determined from the combination of symmetry types that appeared in different organs, such as tentacles, gastric pouches, siphonoglyphs, and muscles [6, 7, 12]. Nevertheless, there is no consensus among studies on which organs define symmetry in anthozoans. As tentacles form from each gastric pouch, the symmetry type for gastric pouch arrangement follows the symmetry type in tentacles. In this study, we employed gastric pouches and muscles as symmetry indicators, as these are common in all anthozoan species.

**Fig. 3:**
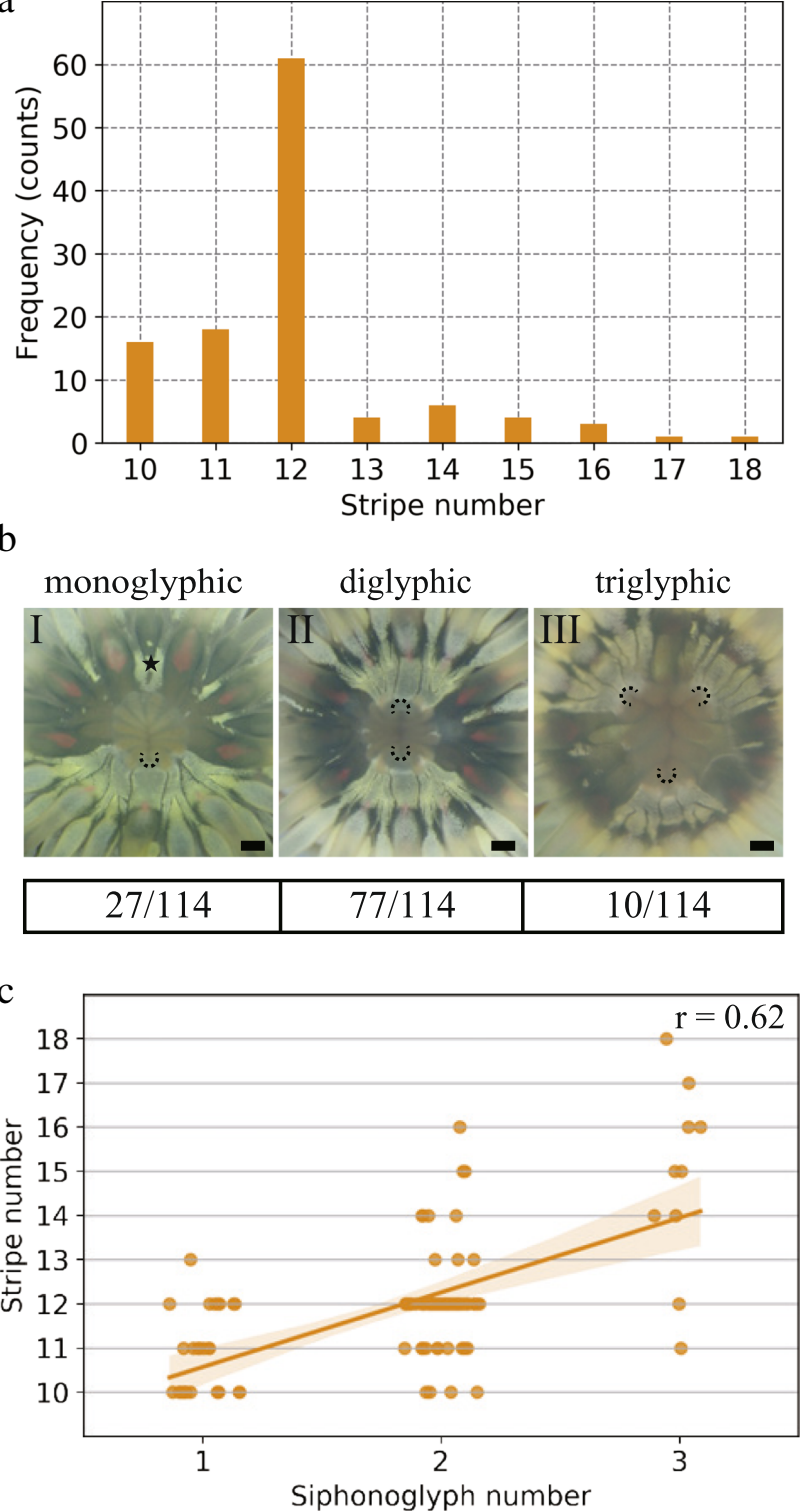
Correlated variation of gastric pouch and siphonoglyph numbers in *D. lineata*. **a** Frequency of individuals as a function of the number of orange stripes corresponding to first, second, and directive gastric pouches. **b** Representative examples (upper panel) and frequency of monoglyphic (I), diglyphic (II), and triglyphic (III) individuals*.* Black dashed arcs indicate siphonoglyphs. Numbers at the bottom of each panel denote the number of individuals, *n* = 114. The star mark indicates intermittent white pigmentation unrelated to the siphonoglyph position. Scale bars, 500 μm. **c** Scatter plot of the stripe number as a function of the siphonoglyph number with the Pearson correlation coefficient $r=\frac{N\left(\Sigma xy\right)-\left(\Sigma x\right)\left(\Sigma y\right)}{\sqrt{\left[N\Sigma {x}^2-{\left(\Sigma x\right)}^2\right]\left[N\Sigma {y}^2-{\left(\Sigma y\right)}^2\right]}}$, where x, y, and Σ denote the number of siphonoglyphs, that of stripes, and the summation for the observed individuals, respectively. Data sets are identical among **a**–**c**

Sea anemones, including *D. lineata*, are formed by two cellular layers (the ectoderm and endoderm). Actiniarian polyps, including those of *D. lineata*, have a single tube-like body structure with tentacles, an actinopharynx in the oral area, and a pedal disk in the aboral area (Fig. 1c). Around the actinopharynx, the gastric cavity is separated into multiple gastric pouches by partitions, referred to as mesenteries (Fig. 1d) [13]. Mesenteries are arranged in couples and classified into three types, i.e., first mesenteries (M1), second mesenteries (M2), and third mesenteries (M3), in decreasing order of length (Figs. 2c, d, g) [14]. Specifically, the M1s reach the actinopharynx, whereas the M2s and M3s do not (Fig. 2d). Directive mesenteries (MDs) [14], positioned on the longitudinal axis of the actinopharynx, are also included in the M1s as long as they reach the actinopharynx (Fig. 2d). Following these definitions, we distinguished the type of mesentery. Here, we referred to each gastric pouch held between a couple of M1s, M2s, M3s, and MDs as a first gastric pouch (P1), second gastric pouch (P2), third gastric pouch (P3), and directive gastric pouch (PD), respectively (Fig. 2d). Each mesentery (M1, M2) has longitudinal retractor muscles arranged at the interior of the gastric pouch, also known as the endocoel side, except for the MDs having muscles at the exterior, also known as the exocoel side (Figs. 2h, i, j, and k). A siphonoglyph is a ciliated groove recognized by a slit-like structure on the actinopharynx (Figs. 2c, g). Specimens with one, two, or three siphonoglyphs are referred to as monoglyphic, diglyphic, and triglyphic individuals (Fig. 3b), respectively [17]. We did not analyze the organ arrangement of triglyphic individuals due to a limited sample number.

### Histological observations and imaging

External morphologies were observed by stereomicroscopy (SZH; Olympus, Tokyo, Japan) and photographed by an Olympus TG-5 (Olympus). For histological sections, samples were relaxed with menthol oil in nursing water [23]. After confirming a lack of tentacle movement, samples were fixed with 4% paraformaldehyde in nursing water, washed, replaced with sucrose solution, and embedded in an optimal cutting temperature compound (Sakura Finetek, Tokyo, Japan). A cryostat (Thermo Fisher Scientific, Waltham, MA, USA) was used to generate 20 μm thick sections. An endogenous red fluorescent protein was overlapped with 4′,6-diamidino-2-phenylindole (DAPI; Roche, Basel, Switzerland) stain to broadly observe the outlines of the mesentery, muscle, and siphonoglyph. A fluorescence microscope (BZ-X700 All-in-one; Keyence, Osaka, Japan) was used to photograph the sections.

### Cloning and expression analyses of fluorescence protein-like genes

To identify fluorescent proteins in *D. lineata*, green fluorescent (GFP)-like proteins were cloned. Total RNA was extracted from the adult samples using Isogen II (NIPPON GENE) following a previously described protocol [24]. These RNAs were reverse transcribed into cDNAs using a GeneRacer Kit (Thermo Fisher Scientific). cDNAs were used as templates in degenerate polymerase chain reaction (PCR), rapid amplification of cDNA (RACE) PCR, and reverse transcription-PCR (RT-PCR). The fragments were amplified by PCR using degenerate primers designed from consensus sequences of the GFP superfamily. Full sequences of the mRNA were determined by RACE PCR using specific primers based on the fragment sequences. The amplified GFP-like genes were subcloned into the pCR Blunt II TOPO vectors (Thermo Fisher Scientific) and verified by sequencing. For protein expression, GFP-like genes containing the transcription start site to end site were amplified by RT-PCR. We identified two clones of fluorescent protein-like genes whose sequences differed by 12 bp. The two clones were subcloned into pETUK (BioDynamics Laboratory Inc., Tokyo, Japan) and expressed in *Escherichia coli* BL21(DE) cells. Fluorescent protein expression in *E. coli* cells was performed according to a previously described protocol [25]. After the incubation and collection of bacteria, soluble protein fractions were isolated from *E. coli* with lysis buffer (Ez Bact Yeast Crusher; Atto Corporation, Tokyo, Japan). The nucleotide and deduced amino acid sequences determined in this study are registered in GenBank, and their accession numbers are listed in Supplementary Table S1.

### Spectroscopy

UV/Vis and fluorescence measurements were both performed with a multichannel flame spectrophotometer (Ocean Insight, Shanghai, China). For UV/Vis absorbance measurements, we utilized a DH-MINI UV-VIS-NIR Fiber Optic Light Source (Ocean Insight). For the excitation source for the fluorescence measurements, a high-intensity blue-light LED (PML2-1005BL light source) was arranged orthogonal to the detector and passed through an LVF-HL linear variable filter (Ocean Insight) so that only light below approximately 480 nm selectively reached the sample. Data visualization and peak wavelength estimation were conducted in R using custom scripts.

### Mathematical model for gastric pouch specification

We derived a model for gastric pouch specification during asexual reproduction, which is regulated by two inhibitory morphogens and one activatory morphogen diffusing in a two-dimensional space of the oral region containing mesenteries, muscles, and siphonoglyphs (Fig. 2d). The gastric pouches were circularly arranged at an equal distance. The spatiotemporal kinetics of the morphogens were represented by the following reaction-diffusion equations:

1 $$ \frac{\partial a}{\partial t}={D}_a\frac{\partial^2a}{\partial {x}^2}+{D}_a\frac{\partial^2a}{\partial {y}^2}-{k}_aa $$

2 $$ \frac{\partial b}{\partial t}={D}_b\frac{\partial^2b}{\partial {x}^2}+{D}_b\frac{\partial^2b}{\partial {y}^2}-{k}_bb $$

and,

3 $$ \frac{\partial c}{\partial t}={D}_c\frac{\partial^2c}{\partial {x}^2}+{D}_c\frac{\partial^2c}{\partial {y}^2}-{k}_cc $$

where *a* denotes the concentration of activator A; *b* and *c* denote the concentrations of inhibitors B and C, respectively; *D*_*a*_, *D*_*b*_, and *D*_*c*_ denote the diffusion coefficients; and *k*_*a*_, *k*_*b*_, and *k*_*c*_ denote the degradation rate. In addition, A is synthesized at PDs with a constant rate *s*_*a*_, whereas both B and C are synthesized at PDs, P1s, and P2s with constant rates of *s*_*b*_ and *s*_*c*_, respectively, which were set differently for PDs (*s*_*b1*_, *s*_*c1*_), P1s (*s*_*b1*_, *s*_*c1*_), and P2s (*s*_*b2*_, *s*_*c2*_) (*s*_*b1*_ > *s*_*b2*_; *s*_*c1*_ < *s*_*c2*_). The parameter values for these equations are reported in Supplementary Table S2. Numerical simulations of the model were performed using the Euler method, a finite difference scheme with first-order approximations of time and space, on a Python-based CompuCell3D platform [26] and under the Neumann boundary condition.

## Results

### *D. lineata* pigmentation at the body wall and oral surface is an externally visible indicator of internal organs

In *D. lineata*, orange-striped pigmentations only appear on the body column at the levels of P1s and P2s (Fig. 2a) [27]. Therefore, we examined whether pigmentation (Figs. 2a, b) could serve as an externally visible indicator of the arrangement of internal organs. Histological analysis confirmed that the orange stripes are associated with all P1s and P2s but not P3s and further revealed that they are restricted to the endodermal cell layer (Figs. 2c, d). As all mesenteries were externally visible through the transparent ectoderm, the mesenteries not adjacent to the orange stripes were identified as M3s (Fig. 2a, d). Moreover, at the oral surface around the actinopharynx, we found fan-shaped white pigmentation (with the same color as the tentacles) at the bases of tentacles surrounding each siphonoglyph (Fig. 2b). Although white pigmentation appeared intermittently in several other places (Fig. 3b) and orange pigmentation weakly appeared at P3 in several individuals (Fig. 2a), these pigmentations could be distinguished from fan-shaped patterns and orange stripes, respectively. Altogether, orange stripes and fan-shaped patterns can serve as external indicators of P1s and P2s, and siphonoglyphs, respectively.

For further histological analyses, we stained the horizontal sections with DAPI to discern the organ outlines (Figs. 2g-k). We revealed that the red fluorescence was always colocalized with orange-striped pigmentations (Figs. 2c, g, Supplementary Fig. S1B), indicating the positions of the P1s and P2s. Red fluorescence was also found at the bases of tentacles initiated between the M1s and M2s (Fig. 2f, Supplementary Fig. S1C). We purified the fluorescent protein from *D. lineata* and found distinctive features of GFP-like proteins; the absorbance and emission peaks were at 561 and 577 nm, respectively, closely resembling the dsRed spectrum (Supplementary Fig. S1A). Thus, the red fluorescence as well as the orange stripes and fan-shaped pattern are externally visible indicators for defining organ arrangement.

### Intraspecific variation in the number of mesenteries and siphonoglyphs with positive correlations

In line with a previous report [17], we confirmed that the number of orange stripes that indicate P1s and P2s varied between 10 and 18, with 12 at the highest frequency (Fig. 3a). In a total of 114 individuals examined, the siphonoglyph number also varied among one (*n* = 27, 24%), two (*n* = 77, 68%), and three (*n* = 10, 8.8%, Fig. 3b), a frequency that was relatively similar to that found in a previous study [17]. Consistently, the stripe numbers in the diglyphic individuals varied from 10 to 16, with a peak at 12 (69% of the 77 samples; Fig. 3c), whereas that in the monoglyphic individuals varied from 10 to 13, with a peak at 10 (44% of the 27 samples), and that in the triglyphic individuals varied from 11 to 18 (Fig. 3c). Thus, we confirmed the intraspecific variation with the predominance of 12 stripes in diglyphic individuals and 10 in monoglyphic individuals. Moreover, we revealed that the siphonoglyph number positively correlated with that of the stripes (Pearson correlation coefficient r = 0.62; Fig. 3c).

### Symmetry polymorphism in the mesenteries and siphonoglyphs

**Fig. 4:**
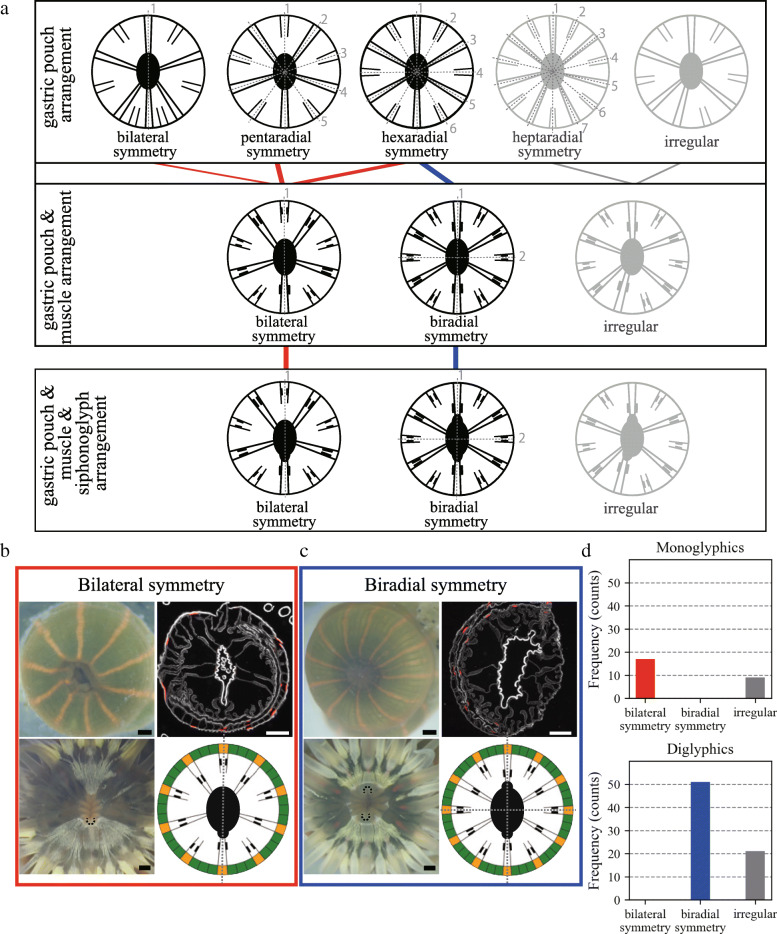
Polymorphic symmetry of internal organ arrangements in correlation with siphonoglyph number. **a** Summary of symmetries in the observed arrangements of gastric pouches alone (upper), those of gastric pouches and muscles (center), and those of gastric pouches, muscles, and siphonoglyphs (lower). The width and color of the lines indicate the number of samples and the symmetry type, respectively (red: bilateral symmetry, blue: biradial symmetry, gray: irregular). **b**, **c** External view (upper left), enlarged view around siphonoglyphs (lower left), horizontal section (upper right), and a schematic view of the corresponding arrangement (lower right). Black dashed arcs indicate siphonoglyphs. Representative individual with bilateral symmetry, a siphonoglyph, and 10 orange stripes (**b**). Representative individual with biradial symmetry, two siphonoglyphs, and 12 orange stripes (**c**). M3s are not shown. Scale bars, 500 μm. Gray dotted lines in A–C indicate symmetry planes. **d** Normalized frequency (%) of bilateral symmetry, biradial symmetry, and irregularity in monoglyphic (upper) and diglyphic (lower) individuals

To examine the variations in body symmetry, we analyzed the positional arrangement of gastric pouches, retractor muscles, and siphonoglyphs (Figs. 2d and 4a). First, the symmetry type in the arrangements of the gastric pouches was analyzed. Since the P1s and P2s were alternately arranged at an equal interval distance in the majority of the individuals (74% of the 98 samples), their arrangement was symmetrical to each plane that passes through the center of the actinopharynx (Fig. 4a, upper). Therefore, the number of symmetry planes was equal to half the sum of the number of P1s and P2s, indicating penta-radial (5-radial), hexa-radial (6-radial), and hepta-radial (7-radial) symmetries for 10-, 12-, and 14-striped individuals, respectively (Fig. 4a, upper). Each of the P3s was positioned at every gap between the P1s and P2s, thereby following symmetry patterns (Figs. 2d, g). The remaining individuals exhibited irregularity in the alternating arrangement (Fig. 4a, upper), e.g., adjacent positioning of two P1s (Supplementary Fig. S2A).

Next, the symmetry in the arrangement of the longitudinal retractor muscles and gastric pouches was analyzed. Since retractor muscles are positioned outside of the PDs and inside of the other gastric pouches [14] (Fig. 2h-k), the arrangement of the PDs is key to distinguishing symmetry (Fig. 4a, center). Indeed, all the 10-striped individuals exhibiting 5-radial symmetry possessed a single PD showing bilateral symmetry, whereas the other pouches were arranged symmetrically to the plane passing through PD and P2 (Fig. 4b). A few 12-striped individuals also possessed a PD showing bilateral symmetry (Supplementary Fig. S4A), in which the PD and P1 were positioned on either side of the symmetry plane. In contrast, most of the 12-striped individuals had two PDs, each placed opposite to its counterpart (Fig. 4c). The other pouches were arranged symmetrically to the plane passing through the PDs and to the other (perpendicular to the former, Fig. 4c). Therefore, the gastric pouch and muscle arrangement indicated biradial symmetry (Fig. 4a, center).

The symmetry was maintained even when the siphonoglyph arrangement was included (Fig. 4a, lower); a PD and a siphonoglyph were colocalized on the same symmetry plane (Fig. 2d). Thus, the diglyphic individuals exhibited biradial symmetry, whereas the monoglyphic individuals exhibited bilateral symmetry (Fig. 4d). Notably, irregular arrangements (i.e., an absence of symmetry) appeared in a fraction of monoglyphic (35%) and diglyphic (29%) individuals, mainly for the gastric pouches (e.g., alternating arrangement of P1 or P2) and occasionally on two siphonoglyphs (e.g., a few individuals with 14 stripes, as shown in Fig. 4a upper panel and Supplementary Figure S2B). We concluded that body symmetry can be determined as either bilateral or biradial depending on the siphonoglyph organ number and arrangement.

### Lateral inhibition and activation model for gastric pouch specification based on observed gastric pouch arrangement

**Fig. 5:**
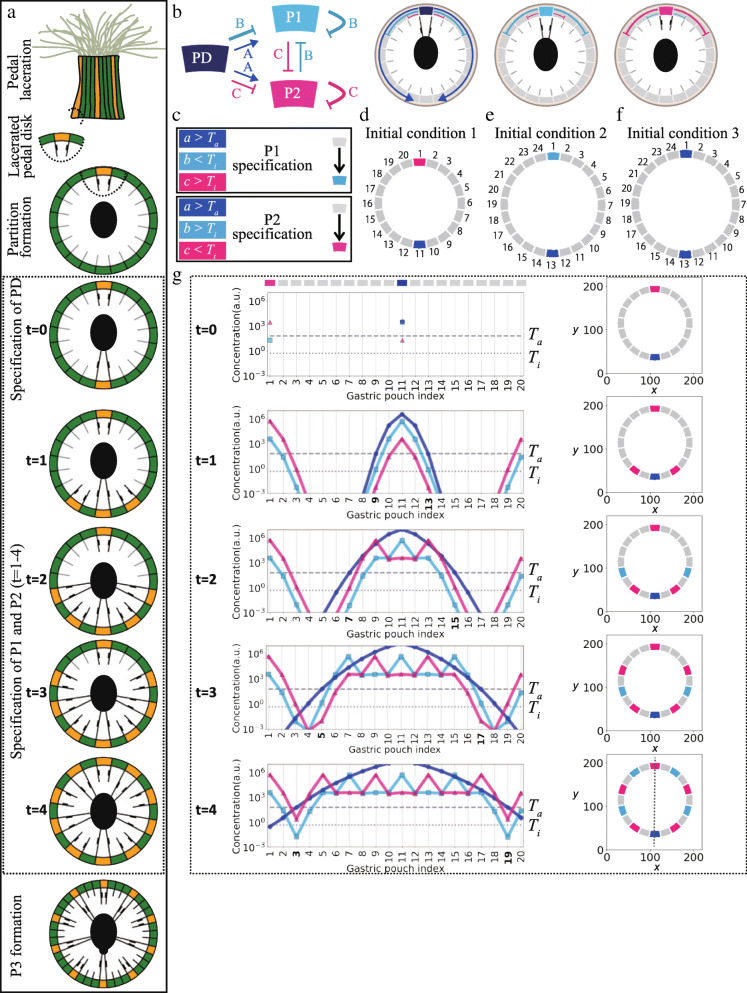
Mathematical model for gastric pouch specification. **a** Schematic diagram of the irreversible specification stages after pedal laceration based on Atoda [28]. Temporal snapshots (denoted by t) framed with dotted lines at the bottom are simulated in our model (Fig. 2d). **b** Regulatory circuit (left) and spatial patterns (right) of an activator (**a**, dark blue) and two inhibitors (**b**, light blue; **C**, magenta). All morphogens are assumed to be diffused at the edge of the endoderm cell layer (light gray region inside) that extends to all oral surfaces but not in the ectoderm (dark gray region outside) or mesoglea (beige region at the center). PD, P1, and P2 are shown in dark blue, light blue, and magenta, respectively. The color of PD was matched with the color of the **a** morphogen, which is secreted only from PD. Colors P1 and P2 were matched up with the colors of the **c** and **c** morphogens, which inhibit the specification of P1 and P2, respectively. **c** Simulated conditions of activator and inhibitor concentrations (left) for the irreversible specification (right) to P1s (upper panel) and P2s (lower panel). *T*_*a*_; activator threshold, *T*_*i*_; inhibitor threshold. **d–f** Initial conditions (t = 0) with two gastric pouches positioned at two opposite sides. For initial condition 1, 20 pouches were arranged at an 18° interval and a radius of 5 (**d**). For initial conditions 2 and 3, 24 pouches were arranged at 15° intervals and a radius of 6 (**e**, **f**). Gastric pouch indexes were numbered from 1 onwards from the uppermost pouch in a clockwise rotation. PD, P1, and P2 denote directive, first, and second gastric pouches, respectively. **g** Bilaterally symmetrical arrangement for 10-striped individuals (20 pouches) initiated the condition (t = 0) of PD and P2 positioned oppositely (**d**). Temporal evolution (represented by t from upper to bottom panels) of the concentrations of **a**, **b**, and **c** (Eqs. 1–3) in a semilogarithmic plot (left panel) as a function of the gastric pouch index (**d**) and 2-D arrangement of specified and nonspecified pouches (right panel). At the top of the uppermost semilogarithmic plot, the arrangement of pouches in 1-D space with their corresponding colors is written in the line of gastric pouch indices in the plot. Dark blue circular, light blue rectangular, and pink triangular markers are used to demonstrate the concentrations of *a, b*, and *c* in gastric pouches, respectively. Bold fonts are used for the corresponding gastric pouch indexes that achieve irreversible specification conditions at *a, b,* and *c* concentrations. The activator threshold (*T*_*a*_) and inhibitor threshold (*T*_*i*_) are indicated as gray dashed and dotted lines, respectively. Following the rule of irreversible specification, pouches neighboring PD remain nonspecified pouches due to the suprathreshold of *b* and *c (a* > *T*_*a*_, *b,c* > *T*_*i*_) (t = 1, gastric pouch index = 10, 12). Adjacent pouches to these pouches, in which the suprathreshold of *a* was achieved, are specified as P2s due to subthreshold *c* (*a* > *T*_*a*_, *b* > *T*_*i*_, *c* < *T*_*i*_) (t = 1, gastric pouch index = 9,13). B and C Secretion from P2s surrounding pouches as nonspecified pouches (*a* > *T*_*a*_, *b,c* > *T*_*i*_) (t = 2, gastric pouch index = 8, 14). Adjacent pouches to these pouches are irreversibly specified as first pouches (P1s) due to the subthreshold of *b* (*a* > *T*_*a*_, *b* < *T*_*i*_, *c* > *T*_*i*_) (t = 2, gastric pouch index = 7, 15). Surrounding pouches are left as nonspecified pouches, and the adjacent pouches are irreversibly specified as P2s due to the subthreshold of c (*a* > *T*_*a*_, *b* > *T*_*i*_, *c* < *T*_*i*_) (t = 3, gastric pouch index = 5, 17). Adjacent pouches to these pouches are irreversibly specified as first pouches (P1s) due to the subthreshold of *b* (*a* > *T*_*a*_, *b* < *T*_*i*_, *c* > *T*_*i*_) (t = 4, gastric pouch index = 3, 19). Gray dotted lines indicate symmetry planes (right bottom panel)

The correlated polymorphism between the siphonoglyph number and symmetry (Fig. 4d) prompted us to speculate a causal relationship between these two factors. Symmetric arrangements of the gastric pouches (Figs. 4b, c) emerged after irreversible specification of permanent pouches to PDs, P1s, or P2s during the asexual reproduction process (Figs. 1e and 5a) [29]. After the specification completes, each nonspecified gastric pouch (Fig. 5a) provides space for the formation of P3 (Figs. 2d and 5a). Since the positioning of the specified gastric pouches sufficiently reflects that of the mesenteries (M1, M2, and M3), muscles, and siphonoglyphs (Figs. 2d, g), spatial patterning of the gastric pouch specification should clarify how symmetry emerges in the arrangement of internal organs. To this end, we built a mathematical model for gastric pouch specification based on the observed arrangements.

After the specification, each of the P1s was arranged in every fourth pouch between nonspecified pouches (Fig. 5a, t = 1–4) [28], suggesting lateral inhibition of the specification to P1s in the long range (thick inhibitory arrows in cyan, Fig. 5b). Likewise, the arrangement of the P2s in every fourth pouch (Fig. 5a; t = 1–4) suggests another lateral inhibition on the specification to P2s in the same range (thick inhibitory arrow in magenta, Fig. 5b). In addition, P1s and P2s were alternately arranged, leaving one nonspecified pouch between each specified pouch, suggesting that the specification of P1s and P2s would be weakly inhibited by the presence of P2s and P1s (Fig. 5a), respectively (thin inhibitory arrows in cyan and magenta, Fig. 5b).

Taken together, these results show there are at least two types of inhibitory effects on the specification of P1s and P2s, which are released from both P1s and P2s. Such lateral inhibition of gastric pouch specification has been previously formulated by assuming that morphogens are secreted from gastric pouches during early anthozoan development [15]. Based on the model and the alternative arrangement in *D. lineata*, we introduced two morphogens (B and C) into our model for the asexual reproduction process (Eqs. 2 and 3). This model assumes that the gastric pouches (P1, P2, and PD) serve as signaling centers. Both B and C are secreted from P1s and P2s, inhibiting the specification of the neighboring nonspecified pouches to P1s and P2s, respectively. The secretion (production) rate of B was higher at P1s than at P2s (*s*_*b1*_ > *s*_*b2*_), and the rate of C was higher at P2s than at P1s (*s*_*c1*_ < *s*_*c2*_).

In addition, the temporal order of specification is different between the early development and the present processes; PDs are specified last in the former but first in the latter, preceding the specification of nonspecified pouches to the other P1s and P2s (Fig. 5a) [28], suggesting an inductive effect of PDs on the specification throughout the body (dark blue arrows in Fig. 5b, left). Accordingly, we additionally introduced lateral activation into the model by assuming that another morphogen (A; Eq. 1) is secreted from PDs and diffuses more broadly than the inhibitors (B and C). When the concentration of activator A exceeds a threshold (*a* > *T*_*a*_; activator threshold in Fig. 5c) and that of either inhibitor B or C is below the other threshold (*T*_*i*_; inhibitor threshold) at a nonspecified pouch, it can be specified (Fig. 5c, left panels). In the case of subthreshold B and suprathreshold C (*b* < *T*_*i*_ and *c* > *T*_*i*_), specification to P2 is selectively sufficiently inhibited, resulting in specification to P1 (left upper panel, Fig. 5c). In contrast, P2 is specified in the opposite case (*b* > *T*_*i*_ and *c* < *T*_*i*_; Fig. 5c, left bottom panel). These specifications occur irreversibly, following observations [29]. Thus, *D. lineata* mesentery arrangements provide a lateral inhibition and activation model representing two inhibitors and an activator for mesentery specification during the asexual reproduction process.

The initial conditions of the present model follow the early stage of asexual reproduction. In many cases, in earlier observations, a lacerated pedal disk (Fig. 1e) includes one stripe (53%, 215 in 402 pedal disks) corresponding to a single pouch of either P1, P2, or PD [17]. In contrast to the gastric pouch, a PD is subsequently specified during reproduction (Fig. 5a, t = 0) [28]. These two initially specified pouches and the total number of gastric pouches appeared to be limited to three combinations in our observations, except for the irregular arrangements (Supplementary Figure S2): a PD and a P2 initially specified in 20 pouches (Figs. 4b and 5d, initial condition 1), a PD and a P1 in 24 pouches (Supplementary Figure S4, Fig. 5e, initial condition 2), and two PDs in 24 pouches (Figs. 4c and 5f, initial condition 3). Importantly, the arrangement of the specified and nonspecified pouches under initial conditions 1 and 2 is bilaterally symmetric, whereas that under initial condition 3 is biradially symmetric. Hence, we adopted the three observed initial conditions with either bilateral or biradial symmetry; two gastric pouches are already specified, whereas the others are nonspecified.

### Siphonoglyph number encodes symmetry through lateral inhibition and activation

To examine whether the lateral inhibition and activation model accounts for the emergence of biradial and bilateral symmetry, we performed model simulations using the three combinations of initial conditions (Figs. 5d–f). We first examined initial condition 1 (Fig. 5d), in which P2 and PD among 20 pouches were already specified on opposite sides (Fig. 5g, t = 0; Supplementary Figure S3A). The pouches adjacent to the PD consistently remained nonspecified due to suprathresholds *b* and *c*, which were secreted from the PD despite suprathreshold *a* being secreted from the PD (Fig. 5g, t = 1, gastric pouch index = 10 and 12). Conversely, the second adjacent pouches were specified irreversibly as P2s due to the subthreshold of *c* (Fig. 5g, t = 1, gastric pouch index = 9 and 13). These P2s subsequently began to secrete inhibitors B and C. These morphogens suppressed the specification of the adjacent pouches due to the suprathreshold of *b* and *c* (Fig. 5g, t = 2, gastric pouch index = 8 and 14) but allowed the irreversible specification of the second neighbors to P1s due to subthreshold *b* and suprathreshold *a* (Fig. 5g, t = 2, gastric pouch index = 7 and 15). While repeating such sequential specification processes, P1s and P2s were alternately specified every two pouches (Fig. 5g, t = 3 and 4). Importantly, the series of specification orders (Fig. 5g, t = 3 and 4) agreed with the observations of a previous study [28], further validating the model. Given an equal diffusion of these morphogens in clockwise and counterclockwise directions (Supplementary Figure S3A), the specification occurred symmetrically to a single plane, passing through the initially specified two pouches (i.e., a PD and a P2), and thereby reproduced the observed arrangement of bilateral symmetry (Figs. 4b and 5g, t = 4).

Moreover, under the second initial condition of 24 pouches, in which P1 and PD are specified on opposite sides (Fig. 5e), our model succeeded in reproducing the other arrangement of bilateral symmetry to the plane passing through the two pouches initially specified (Supplementary Figure S4B). Thus, the arrangements of the specified and nonspecified pouches, starting from the first and second initial conditions with a single PD, developed to the observed arrangements, retaining bilateral symmetry under 20 and 24 pouches through lateral activation and inhibitions.

**Fig. 6:**
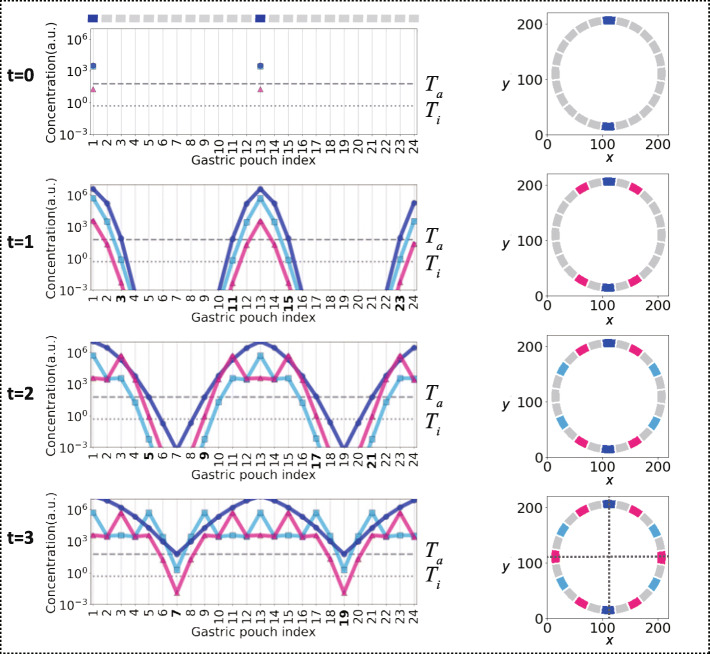
A biradially symmetrical arrangement for 12-striped individuals (24 pouches) in the model simulation. Temporal evolution of the concentrations of **a**, **b**, and **c** in a semilogarithmic plot (left panel) as a function of the gastric pouch index (Fig. 5f) and 2-D arrangement of specified and nonspecified pouches (right panel). Except for the initial condition (t = 0) of the two PDs positioned oppositely (Fig. 5f), the definitions of the colors, lines, and indexes used in each panel and the model setting are identical to those in Fig. 5g. Following the same rule of irreversible specification in Fig. 5g, pouches neighboring two PDs remain nonspecified pouches due to the suprathreshold of *b* and *c (a* > *T*_*a*_, *b,c* > *T*_*i*_) (t = 1, gastric pouch index = 2, 12, 14, 24). Adjacent pouches to these pouches, in which the suprathreshold of *a* was achieved, are specified as P2s due to subthreshold *c* (*a* > *T*_*a*_, *b* > *T*_*i*_, *c* < *T*_*i*_) (t = 1, gastric pouch index = 3, 11, 15, 23). B and C Secretion from P2s surrounding pouches as nonspecified pouches (*a* > *T*_*a*_, *b,c* > *T*_*i*_) (t = 2, gastric pouch index = 4, 10, 16, 22). Adjacent pouches to these pouches are irreversibly specified as first pouches (P1s) due to the subthreshold of *b* (*a* > *T*_*a*_, *b* < *T*_*i*_, *c* > *T*_*i*_) (t = 2, gastric pouch index = 5, 9, 17, 21). Surrounding pouches are left as nonspecified pouches, and the adjacent pouches are irreversibly specified as P2s due to the subthreshold of c (*a* > *T*_*a*_, *b* > *T*_*i*_, *c* < *T*_*i*_) (t = 3, gastric pouch index = 7, 19). Gray dotted lines indicate symmetry planes (right bottom panel)

Under the third initial condition of two PDs being specified among 24 pouches (Fig. 5f), activator A, inhibitor B, and C morphogens were released equally from these two PDs (Fig. 6, t = 0; Supplementary Figure S3B). Therefore, the sequential specification of pouches occurred from their proximal sides (Fig. 6, t = 1) and, more importantly, proceeded in a symmetric manner to both the plane passing through the two initially specified PDs and the other perpendicularly oriented plane. Following the same specification rules mentioned above, the arrangement of specified and nonspecified pouches developed to the observed pouch, retaining the biradial symmetry to the abovementioned two planes under 24 pouches.

Altogether, the lateral activation and inhibition model supported that the number of PDs (i.e., siphonoglyphs) is sufficient to determine either bilateral or biradial symmetry in the observed specification arrangements. This is because the lateral activation and inhibition mediated by their equal diffusion from specified pouches allowed the irreversible specification of nonspecified pouches, retaining the symmetry of their specification arrangements. The bilateral symmetry initiated with a single plane passing through a PD (Fig. 5d, e). Biradial symmetry is initiated in the case of two PDs due to a mirror image of lateral activation and inhibition (Fig. 5f). These theoretical results recapitulate that the polymorphism in the symmetric arrangement of specified and unspecified pouches arises from the variation in siphonoglyphs.

## Discussion

### Symmetry polymorphism arises from variation in the siphonoglyph number

Although biradially and bilaterally symmetrical species are both found in anthozoans (Fig. 1a), the intraspecific coexistence of different symmetries remains elusive. In the present study, we found polymorphisms of biradial and bilateral symmetries in *D. lineata* (Fig. 4). Organ arrangements of the sampled individuals (*n* = 98) were mostly constrained to biradially symmetrical arrangements (52%) and bilaterally symmetrical arrangements (17%) (Fig. 4). Through analyzing *D. lineata*, we found a correlation between symmetry types and siphonoglyph numbers, which has also been found among other anthozoan species [12, 16] (Fig. 4). The mathematical model for internal organ positioning (Fig. 5) proposed that the common developmental mechanism can lead to different symmetrical phenotypes in the case of different numbers of siphonoglyphs in early developmental stages (Fig. 5g and 6a). These results support the view that symmetry polymorphism is based on siphonoglyph number variation. This model accounts not only for the final arrangement (Fig. 5g) but also for the developmental time course starting from an initial condition. Some of the developmental time courses for one of the initial conditions had already been observed [28] (Fig. 5a; Initial condition 1 in Fig. 5d). In vivo time-lapse imaging of a time course for the other initial conditions (Fig. 5e, f) further verified the presented difference in the establishment of bilateral and biradial symmetry.

### Model limitations

The proposed model for asexual reproduction did not incorporate all of the processes required to capture the fundamental mechanism of symmetry polymorphism. One missing process is how the PD is initially positioned at the exactly opposite side of the pedal disk (Fig. 5a, t = 0; Fig. 5d-f). This exact positioning may influence the symmetry of the arrangement of the gastric pouch specification. The other process missing from the proposed model is the growth of the gastric pouch size; it remains unknown whether the growth occurs before (Fig. 5a, t ≤ 0) or during the specification (Fig. 5a, t > 0). A future study incorporating these processes into the model can clarify how exact PD positioning emerges and whether growth in size affects the symmetry of the specified organs. In addition, future research can examine whether the present model applies to sexual reproduction (Fig. 1b) by establishing experimental protocols under laboratory conditions and observing development.

### Comparison of morphological features in *D. lineata* with other anthozoans

Some arrangements, such as biradial and irregular arrangements, are seen not only in *D. lineata* (Fig. 4c, Supplementary Figure S2B) but are in other Anthozoan species [18]. In *D. lineata*, biradial symmetry is identified by an alternative arrangement of six couples of P1s and P2s with polarized muscles, with two siphonoglyphs positioned on the same symmetry plane (Fig. 4c). Interestingly, this biradially symmetrical arrangement is the same as that in the Halcampa developmental stage, which is widely seen across several superfamilies (Actinioidea, Metridioidea, Actinostolidae, and Exocoelactinidae; *Actinia* in Fig. 1a) in Actiniaria [14].

The irregular arrangement seen commonly in *D. lineata* and other species is indicated by the alternative arrangement of seven pairs of P1s and P2s, two siphonoglyphs positioned on the different symmetry planes, and polarized muscles (Supplementary Figure 2B). The abovementioned biradial and irregular arrangements have been reported in *Spongiactis japonica*, a member of the Metridioidea superfamily [18]. These arrangements can be reproduced by our mathematical model, suggesting conserved developmental regulation between *D. lineata* and other species. Further studies are needed to reveal how widely these developmental regulations are conserved in anthozoans.

Regarding the bilaterally symmetrical arrangements, the possession of a single siphonoglyph is common in *D. lineata* and many other Anthozoan species with bilateral symmetry, although the arrangement of mesenteries and muscles exhibits considerable differences. The mesenteries are arranged in couples (e.g., two M1s in Fig. 4b) in *D. lineata*, whereas they are isolated without forming couples in the other species (at a developmental stage referred to as the Edwardsia stage, e.g., as in *Nematostella* in Fig. 1a) [14]. Therefore, the bilateral symmetry of *D. lineata* is distinct from that of other bilaterally symmetrical anthozoans.

### Symmetry polymorphism as a consequence of asexual reproduction

The distinct bilaterally symmetrical arrangements in *D. lineata* are probably specifically produced as a result of asexual reproduction. In addition to bilaterally symmetrical arrangements, asexual reproduction in *D. lineata* and in *S. japonica* further produced biradially symmetrical and irregular arrangements [18]. Atoda [27] suggested that organ arrangement variations at lacerated pedal disks during asexual reproduction cause arrangement and number variations in reproduced *D. lineata* individuals. Thus, the initial arrangement serves as a prepattern of the final internal organ arrangement as the primary cause for the polymorphism (Fig. 5g and 6).

There are several different types of asexual reproduction seen in anthozoan species (e.g., transverse fission in *Nematostella vectensis*) [30]. Which asexual reproduction types, other than the pedal laceration analyzed here, produce the variation in siphonoglyph number remains elusive. It will be interesting to further study which asexual reproduction types and which species produce symmetry polymorphisms. This study will accelerate our understanding of the distribution of symmetry types in anthozoans and whether the polymorphism is the origin of symmetry diversification.

## Conclusions

In this study, we found polymorphisms between bilateral and biradial symmetries in the arrangement of specified gastric pouches in *D. lineata*. Bilaterally symmetrical individuals always exhibited one siphonoglyph, whereas biradially symmetrical individuals contained two siphonoglyphs. The observed arrangements and theoretical model results predict that a common regulatory circuit in the specification process produces different symmetries that are encoded according to the siphonoglyph number during the initial stage.

## Supplementary Information

**Additional file 1: Supplementary Fig. 1.** Identification of a red fluorescent protein (Plum) in *Diadumene lineata* A Absorbance (red) and fluorescence (blue) spectra of Plum. B, C External views (upper left and middle), schematic diagrams (upper right), and histological sections (lower) of the endogenous red fluorescence and DAPI in the endodermal cell layer of gastric pouch walls (B) and at the root of tentacles (C). Black scale bar indicates 1 mm. White scale bar indicates 200 μm. **Supplementary Fig. 2.** Organ arrangements in irregular individuals A, B External views of siphonoglyph and stripe arrangement (left two panels), as well as the horizontal section (middle) and corresponding gastric pouch arrangement (right) of a representative individual with irregularity. Two adjacent first pouches (P1s) arranged in 11-striped individuals (A). Two siphonoglyphs were not arranged oppositely in 14-striped individuals (B). Black dashed arcs indicate siphonoglyphs. Scale bar indicates 500μm. **Supplementary Fig. 3.** Morphogen concentration in 2-D space in model simulations. A 10-striped bilaterally symmetrical individual and B 12-striped biradially symmetrical one. Temporal evolution (represented by t from upper to bottom panels) of the concentration of A (left), B (middle), and C (right) (Equations 1–3) in 2-D space (red-blue colormap shown in legend at the top). The initial conditions (t = 0) are a directive gastric pouch (PD) and a second gastric pouch (P2) positioned oppositely (A; Fig. 5d), and two PDs positioned oppositely (B; Fig. 5f). **Supplementary Fig. 4.** A bilaterally symmetrical arrangement for 12-striped individuals (24 pouches) in model simulation. A External views of siphonoglyph and stripe arrangement (left two panels), as well as the horizontal section (middle) and corresponding gastric pouch arrangement (right). Gray dotted lines indicate symmetry planes. Black dashed arcs indicate siphonoglyphs. B Temporal evolution (represented by t from upper to bottom panels) of the concentration of A, B, and C in 2-D space (red-blue colormap; left panel), semi-logarithmic plot as a function of the gastric pouch index, and the 2-D arrangement of specified and non-specified pouches (right panel) in model simulation (Equations 1–3). Except for the initial condition (t = 0) of a first pouch (P1) and a directive pouch (PD) positioned oppositely (Fig. 5e), the definition of the colors, lines, indexes used in each panel as well as the model setting are identical with those in Fig. 5g. Following the same rule of specification in Fig. 5g, neighboring pouches to two PDs remained as non-specified pouches due to the suprathreshold of *b* and *c (a* > *T*_*a*_, *b,c* > *T*_*i*_) (t = 1, gastric pouch index = 12, 14). Adjacent pouches to these, in which the suprathreshold of *a* was achieved, were specified as P2s due to the subthreshold *c* (*a* > *T*_*a*_, *b* > *T*_*i*_, *c* < *T*_*i*_) (t = 1, gastric pouch index = 11, 15). B and C secretion from P2s left surrounding pouches as non-specified ones (*a* > *T*_*a*_, *b,c* > *T*_*i*_) (t = 2, gastric pouch index = 10, 16). Adjacent pouches to these are specified as first pouches (P1s) due to the subthreshold of *b* (*a* > *T*_*a*_, *b* < *T*_*i*_, *c* > *T*_*i*_) (t = 2, gastric pouch index = 9, 17). Surrounding pouches were left as non-specified ones, and the adjacent pouches were specified as P2s due to the subthreshold of c (*a* > *T*_*a*_, *b* > *T*_*i*_, *c* < *T*_*i*_) (t = 3, gastric pouch index = 7, 19). B and C secretion from P2s left surrounding pouches as non-specified ones (*a* > *T*_*a*_, *b,c* > *T*_*i*_) (t = 4, gastric pouch index = 6, 20). Adjacent pouches to these are specified as first pouches (P1s) due to the subthreshold of *b* (*a* > *T*_*a*_, *b* < *T*_*i*_, *c* > *T*_*i*_) (t =4, gastric pouch index = 5, 21). Surrounding pouches were left as non-specified ones, and the adjacent pouches were specified as P2s due to the subthreshold of c (*a* > *T*_*a*_, *b* > *T*_*i*_, *c* < *T*_*i*_) (t = 5, gastric pouch index = 3, 23). Gray dotted lines indicate symmetry planes (right bottom panel). Scale bar indicates 500 μm. **Supplementary Table 1.** Primers used for degenerate, RACE, and RT-PCR and accession numbers. **Supplementary Table 2.** Parameters used in the mathematical model.

## Data Availability

Relevant data are available from the corresponding author upon reasonable request.

## Abbreviations

me: mesentery
ap: actinopharynx
mu: muscle
sp: siphonoglyph
M1: first mesenteries
M2: second mesenteries
M3: third mesenteries
MD: directive mesenteries
P1: first gastric pouch
P2: second gastric pouch
P3: third gastric pouch
PD: directive gastric pouch
