# Supplementary material for: Polymorphism in the symmetries of gastric pouch arrangements in the sea anemone D. lineata
Source: Zoological Lett. 2021 Sep 6;7:12. doi: 10.1186/s40851-021-00180-0 (PMC8419960; doi:10.1186/s40851-021-00180-0)
Supplement: Supplementary file 1 — Additional file 1: Supplementary Fig. 1. Identification of a red fluorescent protein (Plum) in Diadumene lineata A Absorbance (red) and fluorescence (blue) spectra of Plum. B, C External views (upper left and middle), schematic diagrams (upper right), and histological sections (lower) of the endogenous red fluorescence and DAPI in the endodermal cell layer of gastric pouch walls (B) and at the root of tentacles (C). Black scale bar indicates 1 mm. White scale bar indicates 200 μm. Supplementary Fig. 2. Organ arrangements in irregular individuals A, B External views of siphonoglyph and stripe arrangement (left two panels), as well as the horizontal section (middle) and corresponding gastric pouch arrangement (right) of a representative individual with irregularity. Two adjacent first pouches (P1s) arranged in 11-striped individuals (A). Two siphonoglyphs were not arranged oppositely in 14-striped individuals (B). Black dashed arcs indicate siphonoglyphs. Scale bar indicates 500μm. Supplementary Fig. 3. Morphogen concentration in 2-D space in model simulations. A 10-striped bilaterally symmetrical individual and B 12-striped biradially symmetrical one. Temporal evolution (represented by t from upper to bottom panels) of the concentration of A (left), B (middle), and C (right) (Equations 1–3) in 2-D space (red-blue colormap shown in legend at the top). The initial conditions (t = 0) are a directive gastric pouch (PD) and a second gastric pouch (P2) positioned oppositely (A; Fig. 5d), and two PDs positioned oppositely (B; Fig. 5f). Supplementary Fig. 4. A bilaterally symmetrical arrangement for 12-striped individuals (24 pouches) in model simulation. A External views of siphonoglyph and stripe arrangement (left two panels), as well as the horizontal section (middle) and corresponding gastric pouch arrangement (right). Gray dotted lines indicate symmetry planes. Black dashed arcs indicate siphonoglyphs. B Temporal evolution (represented by t from upper to bottom panels) [file 40851_2021_180_MOESM1_ESM.docx]

**
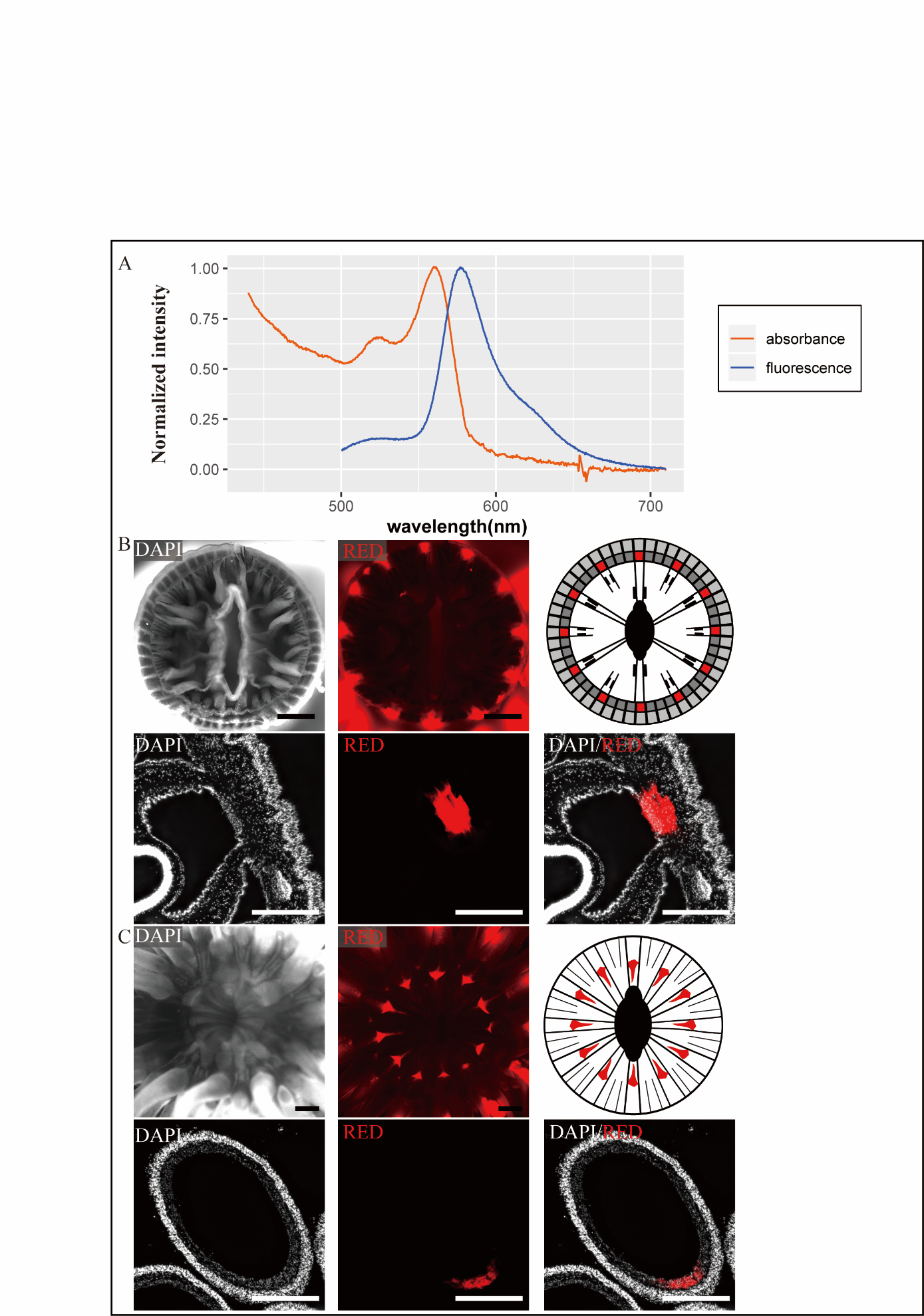
**

**Supplementary Fig. 1** **Identification of a red fluorescent protein (Plum) in *Diadumene lineata* A** Absorbance (red) and fluorescence (blue) spectra of Plum. **B, C** External views (upper left and middle), schematic diagrams (upper right), and histological sections (lower) of the endogenous red fluorescence and DAPI in the endodermal cell layer of gastric pouch walls (B) and at the root of tentacles (C). Black scale bar indicates 1 mm. White scale bar indicates 200 µm.

**
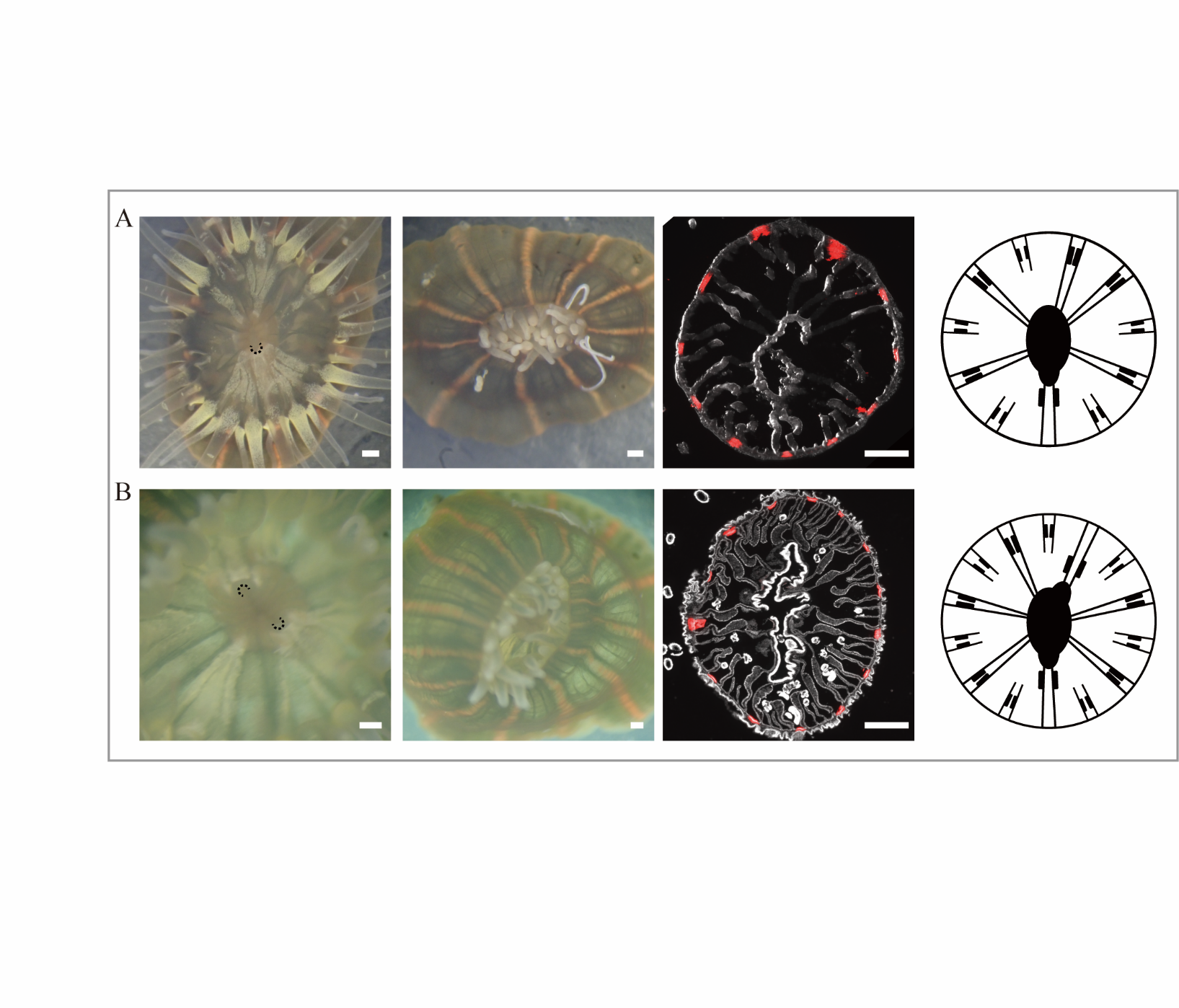
**

**Supplementary Fig. 2 Organ arrangements in irregular individuals A, B** External views of siphonoglyph and stripe arrangement (left two panels), as well as the horizontal section (middle) and corresponding gastric pouch arrangement (right) of a representative individual with irregularity. Two adjacent first pouches (P1s) arranged in 11-striped individuals (A). Two siphonoglyphs were not arranged oppositely in 14-striped individuals (B). Black dashed arcs indicate siphonoglyphs. Scale bar indicates 500µm.


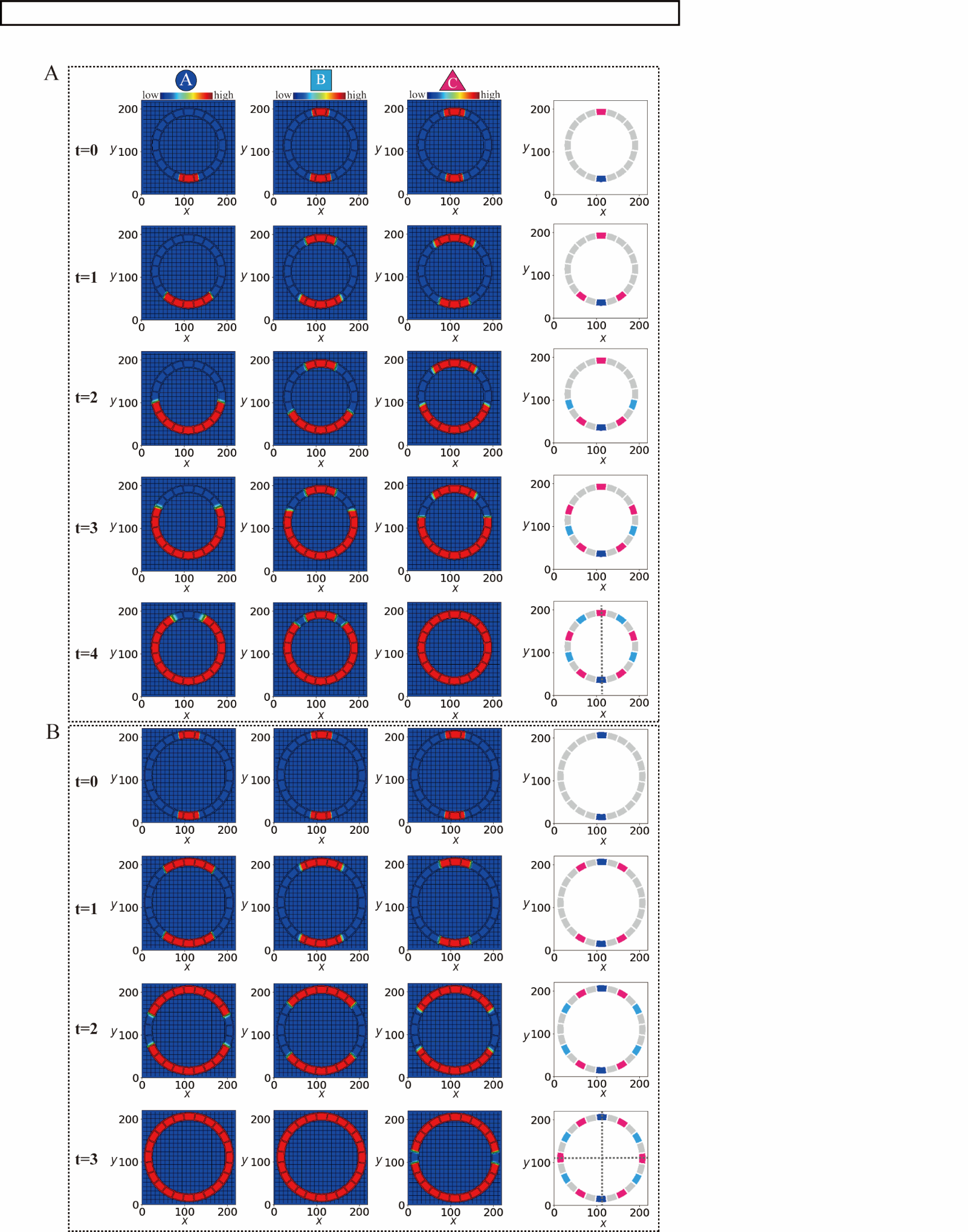
**Supplementary Fig. 3 Morphogen concentration in 2-D space in model simulations**

**A** 10-striped bilaterally symmetrical individual and **B** 12-striped biradially symmetrical one. Temporal evolution (represented by t from upper to bottom panels) of the concentration of A (left), B (middle), and C (right) (Equations 1–3) in 2-D space (red-blue colormap shown in legend at the top). The initial conditions (t = 0) are a directive gastric pouch (PD) and a second gastric pouch (P2) positioned oppositely (A; Fig. 5D), and two PDs positioned oppositely (B; Fig. 5F).

**
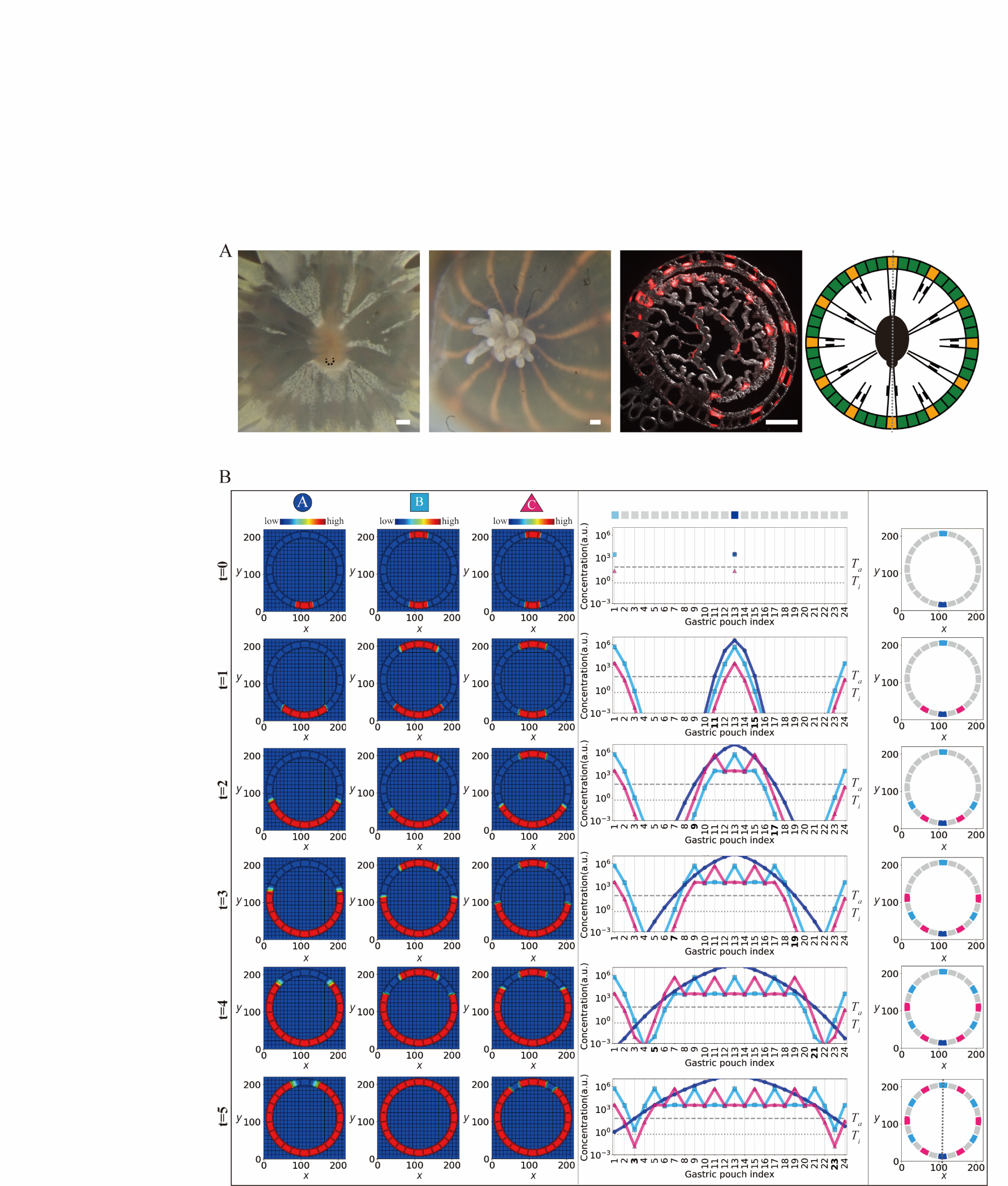
Supplementary Fig. 4 A bilaterally symmetrical arrangement for 12-striped individuals (24 pouches) in model simulation**

**A** External views of siphonoglyph and stripe arrangement (left two panels), as well as the horizontal section (middle) and corresponding gastric pouch arrangement (right). Gray dotted lines indicate symmetry planes. Black dashed arcs indicate siphonoglyphs. **B** Temporal evolution (represented by t from upper to bottom panels) of the concentration of A, B, and C in 2-D space (red-blue colormap; left panel), semi-logarithmic plot as a function of the gastric pouch index, and the 2-D arrangement of specified and non-specified pouches (right panel) in model simulation (Equations 1–3). Except for the initial condition (t = 0) of a first pouch (P1) and a directive pouch (PD) positioned oppositely (Fig. 5E), the definition of the colors, lines, indexes used in each panel as well as the model setting are identical with those in Figure 5G. Following the same rule of specification in Figure 5G, neighboring pouches to two PDs remained as non-specified pouches due to the suprathreshold of *b* and *c* *(a* > *T_a_*, *b,c* > *T_i_*) (t = 1, gastric pouch index = 12, 14). Adjacent pouches to these, in which the suprathreshold of *a* was achieved, were specified as P2s due to the subthreshold *c* (*a* > *T_a_*, *b* > *T_i_*, *c* < *T_i_*) (t = 1, gastric pouch index = 11, 15). B and C secretion from P2s left surrounding pouches as non-specified ones (*a* > *T_a_*, *b,c* > *T_i_*) (t = 2, gastric pouch index = 10, 16). Adjacent pouches to these are specified as first pouches (P1s) due to the subthreshold of *b* (*a* > *T_a_*, *b* < *T_i_*, *c* > *T_i_*) (t = 2, gastric pouch index = 9, 17). Surrounding pouches were left as non-specified ones, and the adjacent pouches were specified as P2s due to the subthreshold of c (*a* > *T_a_*, *b* > *T_i_*, *c* < *T_i_*) (t = 3, gastric pouch index = 7, 19). B and C secretion from P2s left surrounding pouches as non-specified ones (*a* > *T_a_*, *b,c* > *T_i_*) (t = 4, gastric pouch index = 6, 20). Adjacent pouches to these are specified as first pouches (P1s) due to the subthreshold of *b* (*a* > *T_a_*, *b* < *T_i_*, *c* > *T_i_*) (t =4, gastric pouch index = 5, 21). Surrounding pouches were left as non-specified ones, and the adjacent pouches were specified as P2s due to the subthreshold of c (*a* > *T_a_*, *b* > *T_i_*, *c* < *T_i_*) (t = 5, gastric pouch index = 3, 23). Gray dotted lines indicate symmetry planes (right bottom panel). Scale bar indicates 500 µm.


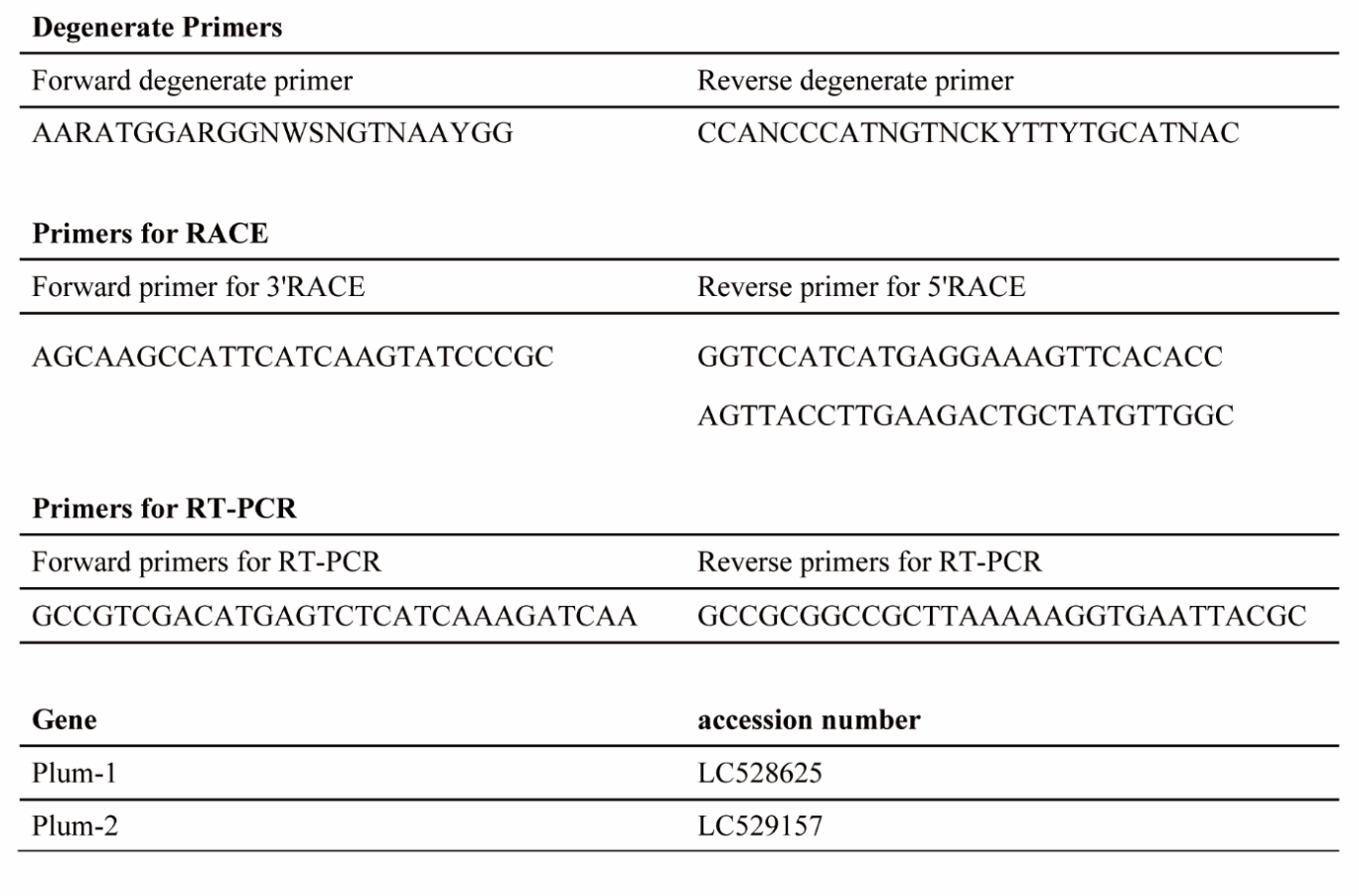
**Supplementary Table 1** Primers used for degenerate, RACE, and RT-PCR and accession numbers

| **parameter** | **value** |
| --- | --- |
| D_a_ | 0.6 |
| D_b_ | 0.7 |
| D_c_ | 0.7 |
| s_a_ | 3220 |
| s_b1_ | 2800 |
| s_b2_ | 20 |
| s_c1_ | 20 |
| s_c2_ | 2800 |
| k_a_ | 8.0x10^-12^ |
| k_b_ | 0.05 |
| k_c_ | 0.05 |
| dt | 0.1 |
| dx  dy | 1  1 |
| T_a_ | 60 |
| T_i_ | 0.5 |

**Supplementary Table 2** Parameters used in the mathematical model
